# Machine Learning to Analyze Factors Associated With Ten-Year Graft Survival of Keratoplasty for Cornea Endothelial Disease

**DOI:** 10.3389/fmed.2022.831352

**Published:** 2022-06-02

**Authors:** Marcus Ang, Feng He, Stephanie Lang, Charumathi Sabanayagam, Ching-Yu Cheng, Anshu Arundhati, Jodhbir S. Mehta

**Affiliations:** ^1^Singapore National Eye Centre, Singapore, Singapore; ^2^Singapore Eye Research Institute, Singapore, Singapore; ^3^Department of Ophthalmology and Visual Sciences, Duke-NUS Medical School, Singapore, Singapore

**Keywords:** machine learning, keratoplasty, graft survival, endothelial (dys)function, penetrating keratoplasty

## Abstract

**Purpose:**

Machine learning analysis of factors associated with 10-year graft survival of Descemet stripping automated endothelial keratoplasty (DSAEK) and penetrating keratoplasty (PK) in Asian eyes.

**Methods:**

Prospective study of donor characteristics, clinical outcomes and complications from consecutive patients (*n* = 1,335) who underwent DSAEK (946 eyes) or PK (389 eyes) for Fuchs’ endothelial dystrophy (FED) or bullous keratopathy (BK) were analyzed. Random survival forests (RSF) analysis using the highest variable importance (VIMP) factors were determined to develop the optimal Cox proportional hazards regression model. Main outcome measure was 10-year graft survival with RSF analysis of factors associated with graft failure.

**Results:**

Mean age was 68 ± 11 years, 47.6% male, in our predominantly Chinese (76.6%) Asian cohort, with more BK compared to FED (62.2 vs. 37.8%, *P* < 0.001). Overall 10-year survival for DSAEK was superior to PK (73.6 vs. 50.9%, log-rank *P* < 0.001). RSF based on VIMP (best Harrell C statistic: 0.701) with multivariable modeling revealed that BK (HR:2.84, 95%CI:1.89–4.26; *P* < 0.001), PK (HR: 1.64, 95%CI:1.19–2.27; *P* = 0.002), male recipients (HR:1.75, 95%CI:1.31–2.34; *P* < 0.001) and poor pre-operative visual acuity (HR: 1.60, 95%CI:1.15–2.22, *P* = 0.005) were associated with graft failure. Ten-year cumulative incidence of complications such as immune-mediated graft rejection (*P* < 0.001), epitheliopathy (*P* < 0.001), and wound dehiscence (*P* = 0.002) were greater in the PK compared to the DSAEK group.

**Conclusion:**

In our study, RSF combined with Cox regression was superior to traditional regression techniques alone in analyzing a large number of high-dimensional factors associated with 10-year corneal graft survival in Asian eyes with cornea endothelial disease.

## Introduction

Corneal transplantation is currently the most frequently performed type of transplant worldwide (1), with corneal endothelial diseases as the leading surgical indication (2). Today, endothelial keratoplasty (EK) has replaced penetrating keratoplasty (PK) as the corneal transplantation of choice for endothelial disease in the United States (3), and increasingly in the rest of the world (4). Currently, Descemet stripping automated endothelial keratoplasty (DSAEK) is the most popular EK technique, supported by eye banks providing pre-cut donor tissue (5). The short-term advantages of DSAEK over PK are related to its minimally invasive approach—avoiding a full-thickness wound that requires sutures, thereby reducing the risk of intraoperative sight-threatening complications, suture-related problems, graft rejection and potential wound dehiscence (6). Thus, faster visual rehabilitation can be achieved, with reduced post-operative corneal astigmatism and potentially superior visual outcomes (7).

However, long-term outcomes of DSAEK compared to the traditional PK in terms of graft survival and complications such as graft rejection still vary in the published literature. Long-term studies from the Asia-Pacific (8, 9) and Europe (10) support the advantages of DSAEK over PK, but national registries in the United Kingdom (11) and Australia (12), have suggested poorer survival outcomes for DSAEK compared to PK for the same indications. While registries reflect “real-world” data from multiple centers with varying surgical techniques and surgeon experience (12), outcomes from such studies are often confounded by differences in donor characteristics or recipient populations, which may be not well delineated (13). Thus, there is an unmet need for long-term studies that directly compare DSAEK and PK outcomes from a variety of populations (14).

A randomized controlled trial is not always feasible to compare DSAEK and PK, and outcomes from registry studies are valuable in providing representative results by including a large number of cases performed by several surgeons (14). However, cornea graft registries often collect a large number of variables generating enormous datasets over time, which can be difficult to analyze using traditional statistical techniques such as Kaplan-Meier survival and Cox proportional hazards regression analyses. Random forests is a machine-learning technique that is gaining popularity to analyze large datasets with less restrictive assumptions, and random survival forests (RSF) can be used to analyze high-dimensional graft survival data (15, 16). This potentially allows us to study a larger number of factors that influence graft survival outcomes with comparable or even better prediction measures. Thus, we used this machine learning method to examine the large database of outcomes prospectively collected from the Singapore Cornea Transplant Registry over 10 years, to examine factors associated with graft failure comparing PK and DSAEK for corneal endothelial diseases.

## Materials and Methods

We collated all the data from our ongoing prospective Singapore Corneal Transplant Study (SCTS) cohort, which tracks all patients who have underwent a cornea transplant through an annual audit (17). Our inclusion and exclusion criteria have been previously described (18), and in this study we included all consecutive patients with either FED or BK who underwent either a primary DSAEK or PK for optical indications, excluding re-grafts and patients requiring systemic immunosuppresion (19). All corneal surgeons from the Singapore National Eye Center performed all surgeries over the same time period (1999–2011), which included cases performed or partially performed by numerous local or international corneal fellows in training under direct supervision. All data collected in this registry audit include patient demographics, diagnosis, details of surgeries including intra-operative complications, pre- and post-operative best-corrected LogMar visual acuity (BCVA), clinical outcomes and post-operative complications (18).

Our main outcome measure was graft survival, where graft failure was defined as irreversible loss of optical clarity, sufficient to compromise vision for a minimum of three consecutive months (20). Complications were monitored and recorded such as primary graft failure, graft rejection, and graft-related infections as previously defined (21). Graft rejection was defined as presence of an endothelial rejection line or inflammation (keratic precipitates, cells in the stroma, or an increase in aqueous cells from a previous visit, with or without any clinically apparent change in recipient stromal thickness or clarity) in the absence of an endothelial rejection line in a previously clear graft. Endothelial cell counts were performed by certified ophthalmic technicians using a non-contact specular microscope (Konan Medical Corp, Hyogo, Japan) as previously decribed (22). Our study followed the principles of the Declaration of Helsinki, with ethics approval obtained from our local Institutional Review Board (SingHealth Centralized IRB, R847/42/2011).

### Surgical Technique

Essentially, PK surgeries were performed using a standard technique previously described (18), with a Hanna vacuum trephine system (Moria Inc, Antony, France). Briefly, the recipient cornea was first excised using the Hanna trephine system. A 0.25- to 0.50-mm oversized donor cornea then was punched out endothelial side up and sutured on to the recipient with 10-0 nylon, using either 8-bite, 10-0 nylon double continuous running suture or a combination of a single 8-bite 10-0 nylon continuous and 8 interrupted sutures. All DSAEK surgeries were performed using pull-through techniques as previously described (23). Donors were prepared by the surgeon or eye bank technician using an automated lamellar therapeutic keratoplasty system (ALTK, Moria SA, Antony, France). Essentially, after recipient Descemets membrane stripping, insertion of anterior chamber (AC) maintainer and preplaced venting incisions, a DSAEK forceps (ASICO, IL, United States) was used to pull the donor cornea through the scleral incision using a sheets glide (BD Visitec) (23), or a donor inserter device (Endoglide, Network Medical Products, North Yorkshire, United Kingdom) (24). An inferior peripheral iridectomy was performed through a limbal stab incision. Wounds were secured with 10/0 nylon interrupted sutures, and a full air tamponade under slight compression was achieved with a large bubble in the AC for varying periods of time, ranging from 2 to 8 min, while removing interface fluid from the venting incisions. For both PK and DSAEK surgeries a bandage contact lens was placed at the end, and dexamethasone (0.1%) (Merck & Co Inc, Rahway, NJ, United States), gentamicin (14 mg/ml, Schering AG, Berlin-Wedding, Germany), and cefazolin (50 mg/ml, GlaxoSmithKline, NC, United States) was injected subconjunctivally after all surgeries. All PK and DSAEK patients received a standard post-operative regime: topical antibiotic (levofloxacin 0.5%, Santen, Osaka, Japan) and topical prednisolone acetate ophthalmic suspension 1% (Allergan, Marlow, United Kingdom) three hourly for a month, four times daily for 2 months, which was tapered by one drop per 3 month down to 1 drop per day dosing by one year, and thereafter continued indefinitely.

### Statistical Analysis

For the current study, 49 variables from SCTS audit were identified by literature review for their potential relevance to the graft failure, including donor and recipient demographics, clinical data (visual acuity, ocular findings, etc.), and operative data (primary procedure, secondary procedure, donor/recipient sizes, surgical complications, etc.) (Supplementary Table 1). We used a RSF machine learning algorithm for multivariate survival analysis to detect important linear, non-linear, and interaction effects among variables (25). These variables were fed into a RSF model consisting of 10,000 trees, where each tree was grown using the log-rank splitting rule on a random sample of 63.2% of the original population by default, with additional RSF parameters (e.g., node size, number of variables to try at each potential split) tuned using a greedy approach to minimize the out-of-bag (OOB) error rate, that is, the error rate using the remaining data not used for model training (25). We then ranked top variables and pair-wise interactions according to their VIMP scores (larger VIMP indicates greater importance for a successful prediction model). Based on VIMP ranking, we then analyzed a sequence of nested Cox regression models using the top 15 variables, among which the model using best variables that achieved the best OOB Harrell C statistic (OOB C-index) will be used. Simply, the OOB C-index is a validation score that estimates the prediction error of random forests (25). Multivariate Cox proportional hazards regression analysis based on this model was used to describe the factors associated with graft failure represented using hazard ratios (HR) and its relative 95% confidence interval (95% CI). Proportional hazard assumption was validated using both individual and global Schoenfeld Test. We used penalized splines from R package survival to assess non-linearity for all continuous variables in the nested Cox regression models. Kaplan–Meier (KM) survival analysis was conducted to compare 10-year survival probabilities of PK and DSAEK groups. Complications were recorded prospectively in our Singapore Cornea Transplant Registry database and represented as a cumulative incidence rate during the follow-up period of 10 years (17). A *P*-value <0.05 was considered statistically significant. The analysis was conducted using R, version 4.0.2 (R Foundation for Statistical Computing) with the *randomForestSRC* package (26, 27).

## Results

**FIGURE 1:**
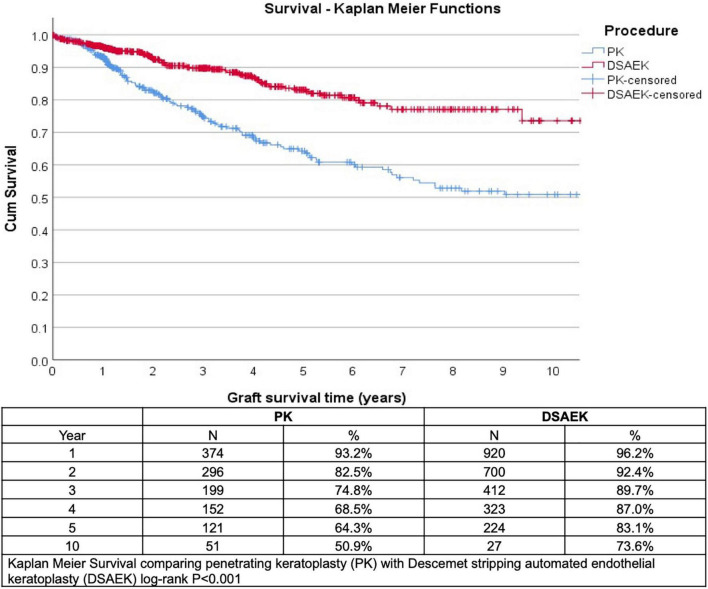
Kaplan–Meier graft survival curves demonstrated superior 5- and 10-year graft survival comparing Descemet stropping automated endothelial keratoplasty (DSAEK) to penetrating keratoplasty (PK), N = number of grafts analyzed (log-rank *P*-value < 0.001).

**FIGURE 2:**
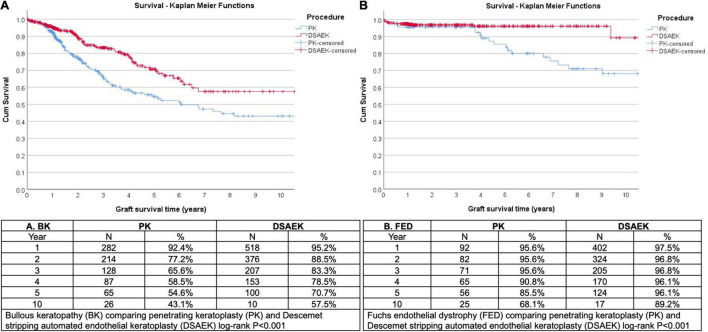
Kaplan Meier graft survival curves demonstrated superior 10-year graft survival comparing Descemet stropping automated endothelial keratoplasty (DSAEK) to penetrating keratoplasty (PK) in eyes (N = number of grafts analyzed) with **(A)** bullous keratopathy (BK, log-rank P < 0.001) and **(B)** Fuchs endothelial dystrophy (FED, log-rank *P* < 0.001).

We analyzed 1,335 consecutive patients who underwent either PK (389 eyes) or DSAEK (946 eyes) based on our inclusion criteria. Overall mean age was 68 ± 11 years, 47.6% male, in our predominantly Chinese (76.6%) Asian cohort with no significant differences in these baseline demographics in our PK and DSAEK groups (Table 1). We had a higher proportion of patients with BK compared to FED (62.2 vs. 37.8%, *P* < 0.001) in our study cohort (Supplementary Table 2). Five-year cumulative graft survival was superior for DSAEK compared to PK (83.1 vs. 64.3%)—log-rank *P* value < 0.001; while 10-year cumulative graft survival was superior for DSAEK compared to PK (73.6 vs. 50.9%)—log-rank *P* value < 0.001 in the remaining surviving grafts (*n* = 78) (Figure 1). Sub-group analysis also revealed significantly superior 10-year survival comparing DSAEK to PK in the BK (57.5 vs. 43.1%, *P* < 0.001) and FED (89.2 vs. 68.1%, *P* < 0.001) groups (Figure 2).

**TABLE 1:** Baseline characteristics of study cohort comparing penetrating keratoplasty (PK) and Descemet stripping automated endothelial keratoplasty (DSAEK) from the Singapore Cornea Transplant Registry.

Characteristics		Corneal Graft		*P* value*
	Total (*n* = 1,335)	PK (*n* = 389)	DSAEK (*n* = 946)	
Mean age, years (± SD)	68.3 ± 11.4	67.4 ± 12.0	68.7 ± 11.1	0.212
**Gender (%)**				
Male	635 (47.6)	191 (49.1)	444 (46.9)	0.509
Female	700 (52.4)	198 (50.9)	502 (53.1)	
**Race (%)**				
Chinese	1,023 (76.6)	306 (78.7)	717 (75.8)	0.515
Malay	63 (4.7)	20 (5.1)	43 (4.5)	
Indian	70 (5.2)	18 (4.6)	52 (5.5)	
Others	179 (13.4)	45 (11.6)	134 (14.2)	
**Surgical indication**				
Fuchs Dystrophy (FED)	504 (37.8)	93 (23.9)	411 (43.4)	< 0.001
Bullous Keratopathy (BK)	831 (62.2)	296 (76.1)	535 (56.6)	
**Baseline/preoperative**				
Visual Acuity (logMAR) (mean, SD)	1.24 ± 0.58	1.57 ± 0.45	1.10 ± 0.57	< 0.001
Endothelial cell counts (cells/mm^2^, SD)	2,819 ± 281	2,704 ± 340	2,865 ± 237	< 0.001

*PK, penetrating keratoplasty; DSAEK, Descemet’s stripping automated endothelial keratoplasty; SD, standard deviation.*

*P value from Mann–Whitney test or chi-square test as appropriate.

**FIGURE 3:**
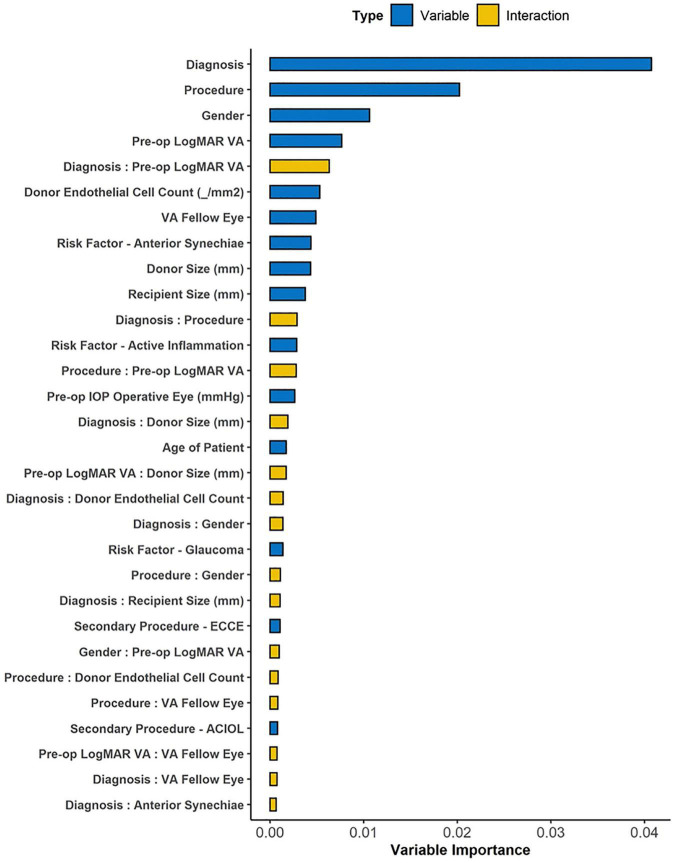
The variable importance (VIMP) plot showing the top 30 variables and interactions for predicting graft failure using Random Survival Forest (RSF) machine learning algorithm. The VIMP score for each candidate variable calculates the difference between the original OOB error rate and the rate after permuting variable values, while VIMP for pair-wise interaction measures the difference between the sum of paired VIMP scores and the VIMP permuting two variables simultaneously. Top-ranked interactions highlight the association between variable pairs that is important for successful prediction in the model.

We ranked top variables and pair-wise interactions according to their VIMP scores (Figure 3) to develop a sequence of nested Cox regression models using the top 15 variables, among which we chose the model with the best variables (diagnosis, procedure, gender, pre-operative visual acuity, and donor endothelial cell count) that achieved the highest OOB C-index of 0.701 on 3,000 bootstrap samples. Using likelihood-ratio tests for nested models, no significant improvement was observed on the model performance after including additional variables (Supplementary Figure 1). Multivariate Cox proportional hazards regression was performed for the top VIMP factors identified by the RSF model that achieved the best OOB Harrell C statistic, i.e., diagnosis (surgical indication, i.e., BK or FED), procedure (surgical technique, i.e., PK or DSAEK), gender, pre-operative visual acuity and donor endothelial cell count was performed (Table 2). We found that BK was a significant factor associated with graft failure (HR: 2.84 95%CI 1.89–4.26; *P* < 0.001) compared to FED and PK was a significant factor associated with graft failure more likely to fail compared to DSAEK (HR: 1.64 95%CI 1.19–2.27; *P* = 0.002).

**TABLE 2:** Hazard ratios for factors associated with 10-year graft failure using random survival forest to determine optimal multivariate regression model.

Factors	N* (*n* = 1,283)	Hazard ratio	*P* > | z|	95% CI	
				Lower	Upper
**Diagnosis/Surgical indication**					
BK	791	2.838	< 0.001	1.892	4.259
FED	492				
**Procedure/surgical technique**					
PK	368	1.643	0.002	1.192	2.265
DSAEK	915				
**Gender**					
Male	608	1.751	< 0.001	1.308	2.344
Female	675				
Pre-operative visual acuity (logMAR)**	1,283	1.601	0.005	1.154	2.220
Donor endothelial cell count**	1,283	1.000	0.171	0.999	1.000

*N = 1283 after excluding 52 subjects who did not have pre-operative visual acuity data available.

**For continuous variables, but linear and non-linear associations were also assessed using penalized splines.

*BK, bullous keratopathy; FED, Fuchs endothelial dystrophy; PK, penetrating keratoplasty; DSAEK, Descemet stripping automated endothelial keratoplasty.*

Overall, we observed a greater 10-year cumulative incidence of complications in the PK compared to DSAEK group (Table 3). Five-year endothelial cell loss was greater in PK compared to DSAEK (67.6 ± 10.7% vs. 53.3 ± 21.0%, *P* = 0.011), as our study was not adequately powered to compare 10-year endothelial cell loss between groups. Complications such as graft rejection (9.5 vs. 4.2%, *P* < 0.001) and corneal epitheliopathy (11.6 vs. 2.5%, *P* < 0.001) were significantly greater in PK compared to DSAEK. There was a greater incidence of transient intraocular pressure (IOP) elevation (as previously defined, i.e., short-term IOP readings > 21 mmHg with ≤ 3 months use of anti-glaucoma medications) comparing PK and DSAEK (26.0 vs. 20.8%, *P* = 0.04). Complications such as wound dehiscence was unique to PK (*P* < 0.001) and graft detachment was unique to DSAEK (*P* < 0.001).

**TABLE 3:** Ten-year cumulative incidence of complications comparing Descemet stripping automated endothelial keratoplasty (DSAEK) and penetrating keratoplasty (PK) in our study cohort.

*Complications	Cumulative incidence (%) ± 95%CI		*P* value
	PK	DSAEK	
Transient elevated IOP (>21 mmHg)	26.0 (21.7–30.6)	20.8 (18.3–23.6)	0.040
Late graft failure	12.3 (9.2–16.0)	4.5 (3.3–6.1)	< 0.001
Epithelial problems	11.6 (8.6–15.2)	2.5 (1.6–3.8)	< 0.001
Graft rejection episode	9.5 (6.8–12.9)	4.2 (3.0–5.7)	< 0.001
Primary graft failure	2.3 (1.1–4.3)	1.8 (1.1–2.9)	0.535
Anterior synechiae	2.3 (1.1–4.3)	1.4 (0.7–2.3)	0.221
Microbial keratitis	2.1 (0.9–4.0)	1.3 (0.7–2.2)	0.281
Wound dehiscence	2.1 (0.9–4.0)	0 (0–0.4)	< 0.001
Cytomegalovirus infection	1.3 (0.4–3.0)	1.4 (0.7–2.3)	0.898
Herpes simplex virus infection	1.0 (0.3–2.6)	0.8 (0.4–1.7)	0.754
Endophthalmitis	0.5 (0.1–1.8)	0.2 (0–0.8)	0.585
Graft detachment	0 (0–0.9)	3.5 (2.4–4.9)	< 0.001

*PK, penetrating keratoplasty; DSAEK, Descemet’s stripping automated endothelial keratoplasty; SD, standard deviation; IOP, intraocular pressure.*

*Complications as recorded in our prospective Singapore Corneal Transplant Registry database.

## Discussion

The Singapore Cornea Transplant Registry prospectively collects a large database of variables and outcomes as an audit of multiple surgeons of various surgical experience, practicing with standardized surgical techniques and post-operative management (28). Traditionally, we have used statistical methods such as Kaplan-Meier survival with the log-rank test to analyze graft outcomes, which is only able to examine only one variable at a time (29). While Cox proportional hazards regression analysis can analyze multiple factors associated with graft survival, the various stepwise (e.g., backward or forward) variable selection methods often lead to well-described limitations (30). Random forests is gaining popularity as a machine learning technique that is able to handle big data with more flexibility in modeling non-linear effects with interactions, for regression and prediction tasks (15, 16). In this current study, we used a RSF model that enabled us to analyze a large number of variables to determine high-importance values, and derive a model with improved prediction performance (OOB C-index of 0.701, compared to traditional Cox regression modeling OOB C-index of 0.576–0.686) (Supplementary Figure 1). However, the advantages of using a machine learning model may come at a cost when it comes to clinical interpretation, due to the complexity of the ensemble tree learning methods. Thus, we presented our results combining features of the robust decision tree ensemble from the random forest, with elements of a Cox proportional hazards regression to explain the factors associated with graft failure in our study (31).

Based on this RSF technique, we found that PK was almost twice as more likely to fail in 10 years compared to DSAEK in the treatment of corneal endothelial diseases, i.e., bullous keratopathy (BK) and Fuchs dystrophy (FED) in our study cohort. Similar to previous studies (32, 33), our 10-year graft survival was superior in DSAEK compared to PK in eyes with BK (57.5 vs. 43.1%, *P* < 0.001) and FED (89.2 vs. 68.1%, *P* < 0.001). These long-term graft survival results reflect the higher proportion of BK compared in FED in our Asian population, as BK was almost three times more likely to be associated with graft failure compared to FED, and BK has been shown to have poorer outcomes in both PK (34–36) and DSAEK (37–39). Another advantage of using the RSF is the ability to examine non-linear associations between various factors and graft failure. A Cox proportional hazards regression analysis assumes a linear relationship between any continuous predictors and an outcome, i.e., graft failure, and thus donor endothelial cell count was not a significant factor (HR: 1.0, *P* = 0.171) after adjusting for other variables. However, the RSF describes a closely associated but non-linear relationship between the donor endothelial cell count and 10-year graft survival in both PK and DSAEK (Supplementary Figure 2).

The RSF analysis also identified recipient gender as an important factor, with the multivariate Cox regression demonstrating that male recipients and those with poorer pre-operative visual acuity are associated with graft failure. A sub-group multivariate Cox regression analysis of our study cohort comparing gender-recipient matched and unmatched subjects revealed a higher risk of 10-year graft failure amongst the gender unmatched (HR: 1.57 95%CI 1.06–2.33, *P* = 0.024) in the PK group but not in the DSAEK group (HR: 0.82 95%CI 0.53–1.276, *P* = 0.382) (Supplementary Table 3). While this observation is consistent with previous large studies on gender matching in penetrating keratoplasty (40), we found male recipients to be still an independent factor associated with 10-year graft failure in the multivariate model, which requires further study. A poor pre-operative visual acuity may suggest presence of more severe corneal decompensation with edema, or underlying factors such as glaucoma or inflammation that could lead to a higher risk of graft failure (41). Our RSF machine learning technique took into account these possible confounders to suggest that poor pre-operative visual acuity was an important, independent factor associated with graft failure. This has useful clinical implications as we may use this as a potential surrogate to counsel patients for risk of graft failure based on their pre-morbid visual acuity and ocular condition.

There are currently few studies that have reported 10-year outcomes of DSAEK, and to our knowledge, no reports that directly compare 10-year outcomes and complication rates of DSAEK to PK from the same study cohort. Moreover, our study used a relatively novel machine learning analysis technique to study a large number of variables while accounting for interactions and non-linear associations with a better prediction compared to traditional model development methods. Another strength is the availability of long-term graft outcomes from registry data, which can vary according to surgeon versus center experience as surgical outcomes are improved by using standardized techniques and post-operative management protocols (42). Compared to a *post hoc* re-analysis of the Cornea Preservation Time Study to specifically examined intra-operative complications, it was reported that surgeon and eye bank factors were the top 2 factors found to be important predictor of graft failure (16). In our study, we found that surgeon experience and surgery performed from earlier years (based on year performed) were not significant factors associated with graft failure on multivariate analysis. Our study also supports the advantages of DSAEK over PK in terms of a lower incidence of complications over a 10-year follow-up, such as epitheliopathy (*P* < 0.001), graft rejection (*P* < 0.001), and as such, less incidence of raised IOP from steroid response as the need for post-operative steroids may be reduced in DSAEK (*P* = 0.04).

However, we recognize the limitations of our study which included the transition of surgical techniques from PK to DSAEK that was introduced in 2006 onward, and the reduction in number of follow-ups at 10 years. We discussed the effect of surgical experience and patient selection in our previous studies, which was mitigated by our standardized protocols and surgical techniques. Thus, we only selected primary grafts for specific corneal endothelial diseases, i.e., Fuchs dystrophy or bullous keratopathy, and previously detailed the transition of proportion of PK toward DSAEK in our study cohort (8). We also acknowledge the differences in our study demographics compared to other reports, in our predominantly Asian population with shallow anterior chambers and a higher proportion of BK compared to FED (5). Despite these limitations common to most registry studies, we believe that our results provide additional evidence to support the trend toward selective lamellar keratoplasty for endothelial diseases. We also recognize the limitations of the RSF analysis used in our study—for example, potentially favoring continuous variables that have more split points (43). Nonetheless, our RSF model selected categorical variables, which further validated these factors’ significance to graft failure. The use of other algorithms such as conditional inference forest may help generate more accurate VIMP scores (43); however, we highlight that the RSF analysis merely serves as a complementary technique to the traditional Cox regression model.

In summary, our study provides long-term graft survival outcomes and cumulative incidence of complications, highlighting the advantages of DSAEK over PK in the treatment of end-stage corneal endothelial decompensation in Asian eyes. We used machine learning techniques to analyze the large registry database collected over a 10-year audit to determine the most important factors associated with graft failure, and used these factors to derive the optimal model for multivariate analysis, which was superior to traditional techniques. A combination of predictive (machine learning) and explanatory (regression) modeling may be a useful way of analyzing large registry datasets to examine cornea graft survival and factors associated with graft failure in future studies, which may then be used to develop a risk prediction model.

## Data Availability Statement

The data analyzed in this study is subject to the following licenses/restrictions: Anonmyized data collected as prospective audit. Requests to access these datasets should be directed to MA, marcus.ang@snec.com.sg.

## Ethics Statement

The studies involving human participants were reviewed and approved by Institutional Review Board (SingHealth Centralized IRB, R847/42/2011). The patients/participants provided their written informed consent to participate in this study.

## Author Contributions

All authors contributed significantly to the development of the study, analysis, and manuscript preparation.

## Conflict of Interest

The authors declare that the research was conducted in the absence of any commercial or financial relationships that could be construed as a potential conflict of interest.

## Publisher’s Note

All claims expressed in this article are solely those of the authors and do not necessarily represent those of their affiliated organizations, or those of the publisher, the editors and the reviewers. Any product that may be evaluated in this article, or claim that may be made by its manufacturer, is not guaranteed or endorsed by the publisher.

## Supplementary Material

Supplementary Figure 1 Boxplot showing the out of bag (OOB) Harrell C Statistic (C-index) of nested models using top variables identified by VIMP. Forward and backward step-wise multivariate Cox regression modeling only achieved OOB C-index of 0.576–0.686 (based on age, gender, race, diagnosis, surgery, glaucoma, donor age).

Supplementary Figure 2 Partial dependence plot showing the adjusted non-linear association between donor endothelial cell count and 10-year graft survival comparing Descemet stripping automated endothelial keratoplasty (DSAEK) and penetrating keratoplasty (PK).
