# Supplementary material for: Machine Learning to Analyze Factors Associated With Ten-Year Graft Survival of Keratoplasty for Cornea Endothelial Disease
Source: Front Med (Lausanne). 2022 Jun 2;9:831352. doi: 10.3389/fmed.2022.831352 (PMC9200960; doi:10.3389/fmed.2022.831352)
Supplement: Supplementary file 1 [file Data_Sheet_1.PDF]

Supplementary Table 1. List of variables used in the Random Survival Forest

Recipient Factors	Clinical Evaluation
Age	Surgical Indication
Gender	Active Infection
Race - Chinese/ Malay/ Indian/ Caucasian/	Active Inflammation
Eye - Left/Right	Anterior Synechiae
Status	Deep Vascularization
Residency	Glaucoma
Dependency/ Care-giver	Lid disease/ Blepharitis
Education level	Ocular Surface Disease
Occupation	Recipient less than 10 years old
Visual Acuity	Repeat Graft
Visual Acuity of Fellow Eye	Superficial Vascularization
Pre-operative intraocular pressure	Previous Intraocular surgery/ Trauma
Diagnosis (FED or BK)	Clinical factors: Others
Surgical Details	Surgical Details: Secondary Procedure
Surgeon - Year of Experience	Anterior chamber intraocular lens (IOL)
Procedure (PK or DSAEK)	Anterior Vitrectomy
Graft Type/ Preparation	Bandage Lens
Donor Culture	Extracapsular cataract extraction
Donor Size (mm)	Intracapsular cataract extraction
Recipient Size (mm)	Iris Fixated IOL
Donor Age	Peripheral Iridectomy
Donor Endothelial Cell Count (/mm^2)	Phacoemulsification
Donor Gender	Removal and exchange of IOL
Donor Status	Scleral-fixated IOL
Intra-Operative Complications	Transscleral Fixated IOL
	Other secondary procedures
