# Supplementary material for: Machine Learning to Analyze Factors Associated With Ten-Year Graft Survival of Keratoplasty for Cornea Endothelial Disease
Source: Front Med (Lausanne). 2022 Jun 2;9:831352. doi: 10.3389/fmed.2022.831352 (PMC9200960; doi:10.3389/fmed.2022.831352)
Supplement: Supplementary file 2 [file Data_Sheet_2.PDF]

Supplementary Table 2: Baseline characteristics of study cohort comparing Fuchs endothelial dystrophy (FED) and bullous keratopathy (BK) from the Singapore Cornea Transplant Registry

Characteristics	Total (n=1335)	Corneal Graft FED (n=504)	BK (n=831)	*P value
Mean age, years (±SD)	68.3 ± 11.4	67.0 ± 10.2	69.1 ± 11.9	<0.001
Gender (%)				
Male	635 (47.6)	185 (36.7)	450 (54.2)	<0.001
Female	700 (52.4)	319 (63.3)	381 (45.8)	
Race (%)				
Chinese	1023 (76.6)	392 (77.8)	631 (75.9)	0.002
Malay	63 (4.7)	35 (6.9)	28 (3.4)	
Indian	70 (5.2)	26 (5.2)	44 (5.3)	
Others	179 (13.4)	51 (10.1)	128 (15.4)	
Procedure				
PK	389 (29.1)	93 (18.5)	296 (35.6)	<0.001
DSAEK	946 (70.9)	411 (81.5)	535 (64.4)	
Baseline/ Preoperative				
Visual Acuity (logMAR) (mean, SD)	1.24 ± 0.58	0.86 ± 0.52	1.46 ± 0.50	<0.001
Endothelial cell counts (cells/mm^2, SD)	2819 ± 281	2847 ± 260	2802 ± 291	0.001

*P value from Mann-Whitney test or chi-square test as appropriate
