# Supplementary material for: Machine Learning to Analyze Factors Associated With Ten-Year Graft Survival of Keratoplasty for Cornea Endothelial Disease
Source: Front Med (Lausanne). 2022 Jun 2;9:831352. doi: 10.3389/fmed.2022.831352 (PMC9200960; doi:10.3389/fmed.2022.831352)
Supplement: Supplementary file 3 [file Data_Sheet_3.PDF]

Supplementary Table 3. Multivariate proportional hazards regression analysis taking into account donor and recipient gender matching for penetrating keratoplasty (PK) and Desscemet automated endothelial keratoplasty (DSAEK)

Multivariate Cox Regression for PK	n	Hazard Ratio	P>|z|	95% CI	
				Lower	Upper
Gender					
Male	178	1.846	0.003	1.235	2.758
Female	190	ref=1			
Diagnosis					
PBK	277	1.979	0.014	1.147	3.414
FED	91	ref=1			
Pre-op VA (logMAR)	368	1.622	0.083	0.938	2.802
Donor ECC	368	1.000	0.098	0.999	1.000
Donor-Recipient Gender					
Unmatched	190	1.574	0.024	1.063	2.331
Matched	178	ref=1			
**Multivariate Cox Regression for DSAEK**	**n**	**Hazard Ratio**	**P>|z|**	**95% CI**	
				Lower	Upper
Gender					
Male	430	1.715	0.020	1.087	2.706
Female	485				
Diagnosis					
PBK	514	4.028	<0.001	2.178	7.449
FED	401				
Pre-op VA (logMAR)	915	1.528	0.046	1.008	2.314
Donor ECC	915	1.000	0.941	0.999	1.001
Donor-Recipient Gender					
Unmatched	479	0.822	0.382	0.529	1.276
Matched	436				
