# Supplementary figures and images for: Machine Learning to Analyze Factors Associated With Ten-Year Graft Survival of Keratoplasty for Cornea Endothelial Disease
Source: Front Med (Lausanne). 2022 Jun 2;9:831352. doi: 10.3389/fmed.2022.831352 (PMC9200960; doi:10.3389/fmed.2022.831352)

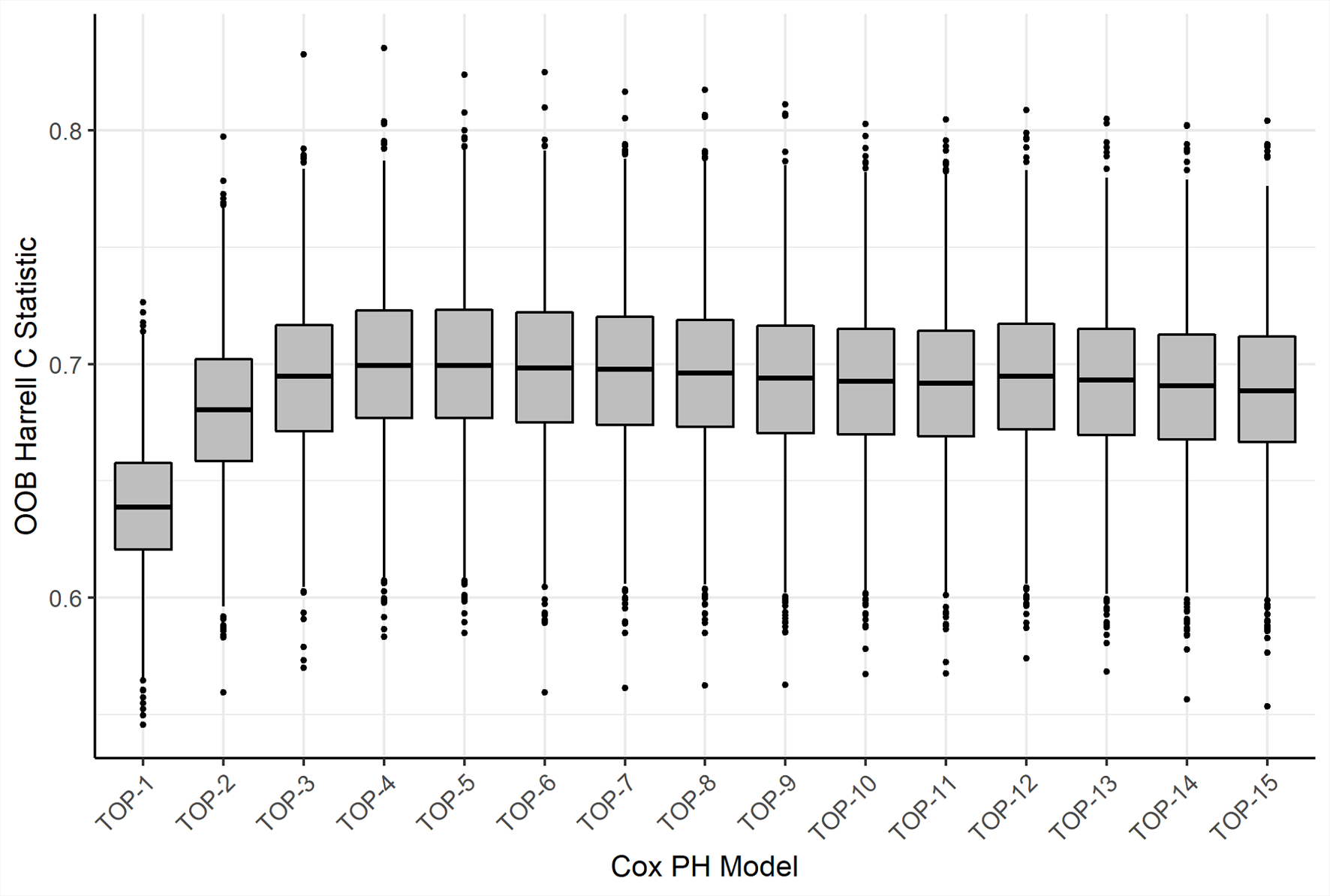

Supplement: Supplementary Figure 1 — Boxplot showing the out of bag (OOB) Harrell C Statistic (C-index) of nested models using top variables identified by VIMP. Forward and backward step-wise multivariate Cox regression modeling only achieved OOB C-index of 0.576–0.686 (based on age, gender, race, diagnosis, surgery, glaucoma, donor age). [file Image_1.TIFF]

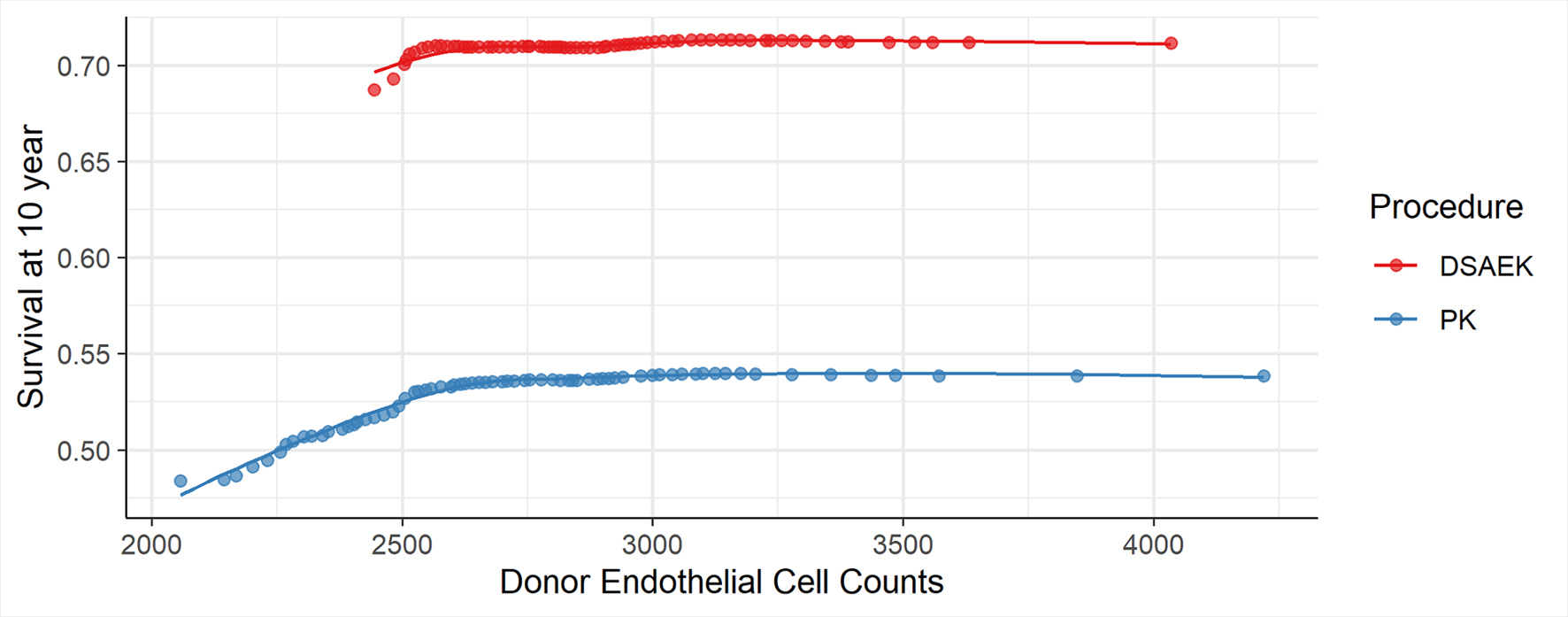

Supplement: Supplementary Figure 2 — Partial dependence plot showing the adjusted non-linear association between donor endothelial cell count and 10-year graft survival comparing Descemet stripping automated endothelial keratoplasty (DSAEK) and penetrating keratoplasty (PK). [file Image_2.TIFF]
